# Thin cutaneous melanoma: immunohistochemical expression of endoglin, VEGF-C and nestin^☆^

**DOI:** 10.1016/j.abd.2021.11.005

**Published:** 2022-09-20

**Authors:** Maria Carolina Widholzer Rey, Adriana Roehe, Felice Riccardi, Beatriz Silva de Souza, Mariele Bevilaqua, Renan Rangel Bonamigo

**Affiliations:** a Department of Dermatology, Universidade Federal de Ciências da Saúde de Porto Alegre, Porto Alegre, RS, Brazil; b Department of Pathology and Forensic Medicine, Postgraduate Program in Pathology, Universidade Federal de Ciências da Saúde de Porto Alegre, Porto Alegre, RS, Brazil; c Hospital Santa Rita, Santa Casa de Misericórdia de Porto Alegre, Executive Director, Grupo Brasileiro de Melanoma, Porto Alegre, RS, Brazil; d Hospital Universitário, Universidade Federal de Santa Catarina, Florianópolis, SC, Brazil; e Santa Casa de Misericórdia de Porto Alegre, Porto Alegre, RS, Brazil

Dear Editor,

The incidence of melanoma is increasing worldwide, and even small, thin tumors can metastasize.^1^, ^2^ Tumor progression includes proliferation, neovascularization, and lymphangiogenesis,^3^ and many mediators are important in this pathogenesis. Nestin (important in proliferation), endoglin (important in neovascularization), and VEGF-C (important in lymphangiogenesis) have already been described in cutaneous melanoma in general; but with little emphasis on thin melanomas.

We describe the clinical data of thin melanomas patients with and without metastases and the immunohistochemical expression of nestin, endoglin, and VEGF-C. The project was approved by the Research Ethics Committee of the institution (Counsel number: 332,405).

A case-control study was carried out with 85 patients: 20 patients in the study group and 65 in the control group.

Patients in the study group were included when they had melanoma metastasis from a thin primary skin lesion (up to 1 mm thick). Controls were patients with thin melanoma but without metastasis. Data were collected regarding sex, age, topography, and histopathological subtype of the tumor.

Of the 85 patients, 37 expressed markers (seven in the study group and 30 in the control group). In the study group, three immunohistochemistry reactions were performed for each marker for each patient. In the control group, two reactions were performed for each marker for each patient, aiming at the statistical analysis of data in clusters. Nestin (ABCAM®), Endoglin (CD105; NOVO CASTRA®) and VEGF-C (IN VITRO GEN®) antibodies were used in the standard immunoperoxidase technique. The chromogenic substance was 3-Amino-9-Ethylcarbazole (AEC), as previously used in a prior study of melanoma.^4^

For endoglin, the lesion hot spots were determined in ×10 magnification fields. Moderate to strong immunohistochemical reactions were considered positive. Up to four hot spots were photographed per slide with ×40 magnification objective. Subsequently, we counted the number of microvessels in each lesion. Areas of fibrosis were avoided, as they could correspond to repair neovascularization instead of neovascularization of the lesion.

To evaluate the differences among the immunohistochemical markers, an analysis based on the Generalized Estimating Equations (GEE) method was used. This technique takes into account the effect of data in clusters, in which a given patient can contribute with a greater number of observations than another. Thus, an adjustment is made for the correlated measures obtained from the same individual. According to the assumptions of data distribution, binding functions per identity (Gaussian), log and Poisson were used.

Regarding the statistical analysis, the quantitative data were described by means and standard error and the categorical variables by counts and percentages. Initial comparisons between groups were performed using Student’s *t* test or Fisher’s exact test. The level of significance was set at α = 0.05; data were entered into an Excel spreadsheet and analyzed using the IBM-SPSS program, version 22.0.

Most patients in the study group were male (75%), whereas, in the control group, 47% of the patients were male. This difference was statistically significant (p = 0.041). The mean age in the study group was 52 years and in the control group, 55 years. In both groups, the most frequent topographic location of the primary melanoma was the trunk. Also in both groups, the most frequent histopathological subtype was superficial spreading melanoma. These data showed no statistical difference (Table 1).

**Table 1:** Patients general characteristics of controls (melanoma without metastasis) and Cases (melnoma with metastasis).

Characteristic	Controls (n = 65)	Cases (n = 20)	p
Age, years	55.0±11.0	52.8±15.9	0.577
Male sex, number (%)	31 (47.7)	15 (75)	0.041
Topography, number (%)			0.209
Trunk	25 (38.5)	14 (70.0)	
Upper limbs	18 (27.7)	2 (10.0)	
Lower limbs	7 (10.8)	2 (10.0)	
Face	5 (7.7)	0	
Scalp	3 (4.6)	1 (5.0)	
Others	7 (10.8)	1 (5.0)	
Histopathological subtype, number (%)			0.311
Superficial spreading melanoma	56 (86.2)	16 (80.0)	
Lentigo malignant melanoma	6 (9.2)	1 (5.0)	
Nodular melanoma	1 (1.5)	1 (5.0)	
Acral melanoma	1 (1.5)	1 (5.0)	
Others	1 (1.5)	1 (5.0)	

Data presented as means ± standard error or counts (percentages).

The immunohistochemical reactions of the markers are shown in Fig. 1 (A: Nestin, B: Nestin, C: Endoglin, D: Endoglin, E: VEGF-C, F: VEGF-C).

**Figure 1:**
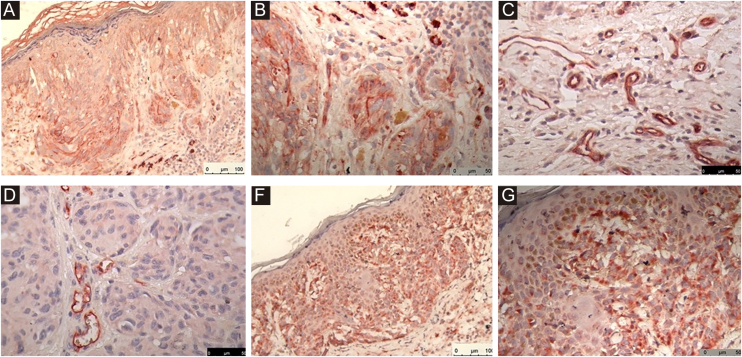
Immunohistochemical reactions: A: nestin (20X), B: nestin (40X), C: endoglin (40X), D: endoglin (40X), E: VEGF-C (20X), F: VEGF-C (40X). The chromogen used was 3-amino-9-etilcarbazol (AEC).

An immunohistochemical reaction was considered positive for endoglin when moderate to strong. In the study group, the mean number of identified vascular structures was 11.47, with a standard error of 1.27. In the control group, it was 9.22, with a standard error of 0.59. This non-significant difference reached a p-value of 0.088. Nestin and VEGF-C showed no differences between the groups.

The male sex is already known to have a worse prognosis among melanoma patients.^2^ In this study, patients with thin melanoma and metastases were significantly more often male than female.

Nestin, important in cell proliferation, and VEGF-C, important in lymphangiogenesis, have been previously described in cutaneous melanoma in general.

Endoglin (also known as CD105) participates in angiogenesis through endothelial cell proliferation and migration and capillary morphogenesis.^5^ The possibility of the involvement of endoglin in the regulation of the biological properties of melanoma cells has been confirmed.^6^ Endoglin expression has been previously detected in melanocytic lesions and in melanoma cell cultures,^7^ indicating a possible involvement of endoglin in the regulation of the biological properties of melanoma cells.^6^

It is possible that endoglin participates in the angiogenesis observed in thin melanomas, especially in tumors with metastases, but this study did not provide definitive statistical evidence.

## Financial support

None declared.

## Authors’ contributions

Maria Carolina Widholzer Rey: Design and planning of the study; data collection/analysis and interpretation of data; statistical analysis; drafting of the manuscript or critical review of important intellectual content; collection, analysis and interpretation of data; intellectual participation in the propaedeutic and/or therapeutic conduct of the studied cases; critical review of the literature; approval of the final version of the manuscript.

Adriana Roehe: Data collection/analysis and interpretation of data; collection, analysis and interpretation of data; effective participation in research orientation; critical review of the literature; approval of the final version of the manuscript.

Felice Riccardi: Data collection/analysis and interpretation of data; collection, analysis and interpretation of data; intellectual participation in the propaedeutic and/or therapeutic conduct of the studied cases; critical review of the literature; approval of the final version of the manuscript.

Beatriz Silva de Souza: Data collection/analysis and interpretation of data; collection, analysis and interpretation of data; critical review of the literature; approval of the final version of the manuscript.

Mariele Bevilaqua: Drafting of the manuscript or critical review of important intellectual content; critical review of the literature; approval of the final version of the manuscript.

Renan Rangel Bonamigo: Design and planning of the study; data collection/analysis and interpretation of data; statistical analysis; drafting of the manuscript or critical review of important intellectual content; collection, analysis, and interpretation of data; effective participation in research orientation; intellectual participation in the propaedeutic and/or therapeutic conduct of the studied cases; critical review of the literature; approval of the final version of the manuscript.

## Conflicts of interest

None declared.
